# Shallow methylmercury production in the marginal sea ice zone of the central Arctic Ocean

**DOI:** 10.1038/srep10318

**Published:** 2015-05-20

**Authors:** Lars-Eric Heimbürger, Jeroen E. Sonke, Daniel Cossa, David Point, Christelle Lagane, Laure Laffont, Benjamin T. Galfond, Marcel Nicolaus, Benjamin Rabe, Michiel Rutgers van der Loeff

**Affiliations:** 1 Geosciences Environment Toulouse (GET), Observatoire Midi-Pyrénées (OMP), UMR CNRS 5563, UMR IRD 234, 14 avenue Edouard Belin, Université Paul Sabatier, 31400 Toulouse, France; 2 ISTerre, Université Joseph Fourier, BP 53, 38041 Grenoble, France; 3 University of Miami Rosenstiel School of Marine & Atmospheric Science, Miami, Florida 33149 USA; 4 Alfred-Wegener-Institut Helmholtz-Zentrum für Polar- und Meeresforschung, 27570 Bremerhaven, Germany

## Abstract

Methylmercury (MeHg) is a neurotoxic compound that threatens wildlife and human health across the Arctic region. Though much is known about the source and dynamics of its inorganic mercury (Hg) precursor, the exact origin of the high MeHg concentrations in Arctic biota remains uncertain. Arctic coastal sediments, coastal marine waters and surface snow are known sites for MeHg production. Observations on marine Hg dynamics, however, have been restricted to the Canadian Archipelago and the Beaufort Sea (<79°N). Here we present the first central Arctic Ocean (79–90°N) profiles for total mercury (tHg) and MeHg. We find elevated tHg and MeHg concentrations in the marginal sea ice zone (81–85°N). Similar to other open ocean basins, Arctic MeHg concentration maxima also occur in the pycnocline waters, but at much shallower depths (150–200 m). The shallow MeHg maxima just below the productive surface layer possibly result in enhanced biological uptake at the base of the Arctic marine food web and may explain the elevated MeHg concentrations in Arctic biota. We suggest that Arctic warming, through thinning sea ice, extension of the seasonal sea ice zone, intensified surface ocean stratification and shifts in plankton ecodynamics, will likely lead to higher marine MeHg production.

The majority of humans are exposed to toxic MeHg *via* the consumption of marine fish^1^. The risk of MeHg exposure is exacerbated for native Arctic populations due to their dependence on marine fish and mammals for protein intake. Marine organisms in the Arctic show elevated MeHg concentrations, which are believed to derive largely from atmospheric deposition of inorganic Hg^2^. There is substantial evidence that Hg deposition to remote locations has increased threefold since pre-industrial times^3^ and much is known about the transport of lower-latitude industrial inorganic Hg emissions to the Arctic^4^ and intense atmospheric Hg deposition events related to sea ice^5^. However, a recent three-dimensional coupled atmosphere-ocean model suggests that the major inorganic Hg source to the Arctic Ocean is provided instead by arctic rivers during spring freshet^4^. While direct evidence is lacking, the magnitude of the spring flood Hg pulse, mainly from Siberian Rivers is under debate^6^. Most importantly, the chain of events that transforms natural and anthropogenic inorganic Hg into toxic bioaccumulating MeHg remains ill-understood^7^.

While Hg measurements of Arctic marine biota are numerous, MeHg observations in sea water of the central Arctic Ocean are inexistent because of analytical and logistical constraints^2^. Despite early work on methylated forms of Hg in the open ocean^8^, fish MeHg has long been thought to originate from MeHg production in coastal and shelf sediments that is advected and bioadvected into open ocean food webs^9^. An incubation study of isotopically labelled Hg species in Arctic coastal sea water shows the potential for *in situ* methylation^10^. Furthermore, observations on the Canadian Archipelago^10^,^11^,^12^ and the Beaufort Sea^13^ suggest that *in situ* methylation in sea water is indeed a relevant phenomenon. MeHg profiles in the Atlantic^14^,^15^, Pacific^1^,^8^, and Southern^16^ Oceans and in the Mediterranean Sea^17^ show maxima in the sub-surface waters^7^. These findings and recent results on Hg isotopic signatures of marine fish^18^ strongly suggest that *in situ* Hg methylation in oxygenated sea water is a potentially dominant source of MeHg to Arctic marine food webs.

In this study we explore for the first time marine tHg and MeHg dynamics in the central Arctic Ocean. The research vessel Polarstern sailed to the North Pole during the TransArc ARK XXVI/3 cruise^19^,^20^ in summer 2011. MeHg refers here to the sum of monomethylmercury (MMHg) and dimethylmercury (DMHg). Four high-resolution unfiltered tHg and MeHg vertical profiles were sampled at locations between 79°N and 90°N (Fig. 1): the coastal influenced open water Laptev Sea station 79°N (PS78/280), the Amundsen Basin station 81°N (PS78/273) at the sea ice edge, the >75% sea ice covered Makarov Basin station 85°N (PS78/245), and the permanently sea ice-covered North Pole station 90°N (PS78/218).

tHg concentrations range from 0.45 to 7.0 pM (0.97 ± 0.76 pM, n = 81). The highest concentration (7.0 pM) is associated with a surface water sample (10 m-depth) at the southernmost station 79°N. Those surface waters are warmer and less salty, indicative of river inputs from Siberia (Fig. S1). The tHg value of 7.0 pM is similar to what has been observed for the Lena River estuary^21^. This observation possibly confirms transfer of arctic river Hg inputs far into the open Arctic Ocean as recently suggested by a three-dimensional numerical Hg model^4^. All waters below that river tongue at the same Laptev Sea station 79°N show low and uniform tHg concentrations (0.53 ± 0.06 pM, n = 21, Fig. 1). The low tHg concentrations may be the result of efficient scavenging by sinking organic matter that originates from the siberian rivers or from enhanced primary production on the siberian shelf^22^,^23^. It has been suggested that scavenging at continental margins can effectively remove tHg^24^. Recent observations in the Beaufort Sea show similar low tHg concentrations at the margin (0.59 pM at 950 m-depth, St 421)^13^. We also observe low tHg concentrations at the North Pole station 90°N (0.54 ± 0.09 pM, n = 12). The North Pole is covered by varying proportions of predominantly multi-year ice and some first-year ice. Surface waters at the North Pole have not been in contact with the atmosphere for several years, and therefore have not received recent inputs from direct atmospheric deposition^25^. North Pole waters may have been stripped of their initial tHg content *via* phytoplankton blooms^23^ (before flowing under the multi-year ice), sinking ice-algae^26^ and/or particle fallout from transpolar drift ice. Alternatively, shelf influenced deep water containing low Hg concentrations (Laptev Sea station 79°N) may have been advected poleward following the general circulation pattern^27^ (Fig. 1). Likely a combination of several factors is causing the low tHg at North Pole.

Stations 81°N and 85°N are located in two distinct gyres in the marginal sea ice zone and show higher tHg concentrations (81°N: 1.3 ± 0.23 pM, n = 22; 85°N: 1.0 ± 0.25 pM, n = 27, Fig. 1). Surface enrichments in tHg (81°N: 2.5 pM and 85°N: 1.7 pM) followed by a gradual decrease with depth suggest surface inputs from melting sea ice, atmospheric precipitation, or rivers. Station 81°N is located at the deepest part of the Gakkel Ridge (recorded bottom depth = 5216 m). Here, waters below 3000 m are trapped in a funnel shaped deep trench. A gradually increasing tHg profile within the trench, to values of 1.5 pM, suggests a small bottom Hg source. The Gakkel Ridge is the world’s slowest spreading ridge and hydrothermal inputs should be of minor importance^28^ (temperature and salinity are relatively uniform within the trench and do not indicate hydrothermal inputs at the time of sampling, Fig. S1). Nevertheless a slow diffusive Hg flux from sediments may be at play. Apart from the peculiar deep features at station 81°N, both the 81°N and 85°N profiles converge to tHg values of 1.0 ± 0.14 pM (n = 36, 200 - 3000 m-depth mean, Fig. 1). While this is twice as high as for the aforementioned stations at 79°N and 90°N, this value is in the range of the North Atlantic Waters flowing into the Arctic Ocean^14^,^15^, which are believed to be enriched with anthropogenic Hg^29^.

At both stations 81°N and 85°N low surface water MeHg levels (81°N: 0.029 pM; 85°N: 0.034 pM), steeply and linearly increase with depth to reach maxima in the shallow pycnocline (81°N: 0.365 pM at 150 m-depth; 85°N: 0.339 pM at 200 m-depth), after which concentrations decrease with depth (Fig. 1). In contrast, our results show also that the stations 79°N and 90°N not only have very low tHg but also remarkably low MeHg concentrations (79°N: 0.025 ± 0.030 pM, n = 22; 90°N: 0.053 ± 0.033 pM, n = 12). This suggests that the lack of supply of inorganic Hg substratum possibly limits MeHg production^30^. The idea has been put forth that sinking organic matter derived from phytoplankton blooms delivers both inorganic Hg and a carbon source to methylating bacteria at depth^8^. In the Arctic, an additional Hg and carbon source may be provided by sea-ice algae^31^,^32^. Several recent studies have found maximum MeHg concentrations in sub-surface global ocean waters where bacterial activity is important^1^,^10^,^11^,^12^,^13^,^14^,^15^,^16^,^17^. An alternative explanation to the *in situ* MeHg production would be that the observed MeHg maxima are an advected feature that has its origin on the continental shelves^9^. However, the North Atlantic Water below the Arctic pycnocline (>200 m-depth) has a residence time of several decades^25^,^27^, while the half-life of marine MeHg against (a)biotic breakdown is relatively short^7^,^10^. Therefore, the combination of low MeHg concentrations at station 79°N closest to the Siberian Shelf and the unlikeliness of long-range advective transport of coastal MeHg suggests that MeHg at stations 81°N and 85°N is produced *in situ* in the pycnocline waters. Stations 81°N and 85°N are both similarly elevated in tHg (means given above) and MeHg profiles (81°N: 0.157 ± 0.103 pM, n = 22; 85°N: 0.210 ± 0.080 pM, n = 27, Fig. 1). Surface waters at both stations show very low MeHg levels, likely due to photodemethylation, biological uptake and evasion to the atmosphere^12^. Station 81°N is located at the sea ice edge and was fully open water during sampling. Station 85°N is also located within the marginal sea ice zone. The sea ice here consists mainly of first-year sea ice and satellite imagery shows substantial open leads before sampling, with sea ice concentration of >75% (Fig. S2). The presence of major open leads at station 85°N could have stimulated primary production in the weeks before sampling, and massive phytoplankton blooms are known to occur under thin first-year sea ice^23^.

One of the most striking features of the MeHg profiles in the marginal sea ice zone (station 81°N and 85°N) is that the MeHg maxima are very shallow (150 – 200 m) compared to other open ocean profiles^7^ (North Atlantic ~ 1000 m, North Pacific ~ 400 - 1000 m, Mediterranean Sea ~ 400 m, Southern Ocean ~ 500 m). In the Arctic Ocean, cold and fresh waters of the polar mixed layer sit on top of the warm and salty Atlantic waters (Fig. S1), generating a strong and shallow halocline (150 - 200 m), which is also the pycnocline^27^. We suggest that sinking particles are slowed down at the shallow pycnocline, undergo remineralization, as also indicated by nutrient profiles^20^ (Fig. S1) and stimulate *in situ* MeHg production. Our high resolution profiles reveal that arctic MeHg maxima occur in high oxygen waters (>290 μM, Fig. S1), and are located deeper than the apparent oxygen utilization (AOU) maxima in the halocline (81°N: 48 μM at 110 m-depth; 85°N: 58 μM at 103 m-depth, Fig. S1). The halocline AOU maximum is generally most dominant throughout the Canadian Basin, and is believed to be largely produced on the shelf^33^. The fact that the shelf generated AOU and MeHg maxima do not collocate also argues for *in situ* MeHg production, rather than an advected feature. Despite lower temperature and higher oxygen concentrations relative to low latitude oceans, we find similar concentrations and fractions of MeHg. Peaks of highest MeHg fraction collocate with the MeHg peaks (81°N: 30% at 150 m-depth; 85°N: 33% at 200 m-depth, Fig. S1), and remain elevated throughout the warm, and salty Atlantic layer.

MeHg bioaccumulation factors are largest at the base of the marine food web, where phytoplankton concentrates sub-picomolar levels of dissolved MeHg to micromolar *in vivo* MeHg levels^7^. Unlike other oceans, the MeHg maxima we observe in the marginal sea ice zone of the Arctic Ocean are located just below the productive surface layer^2^,^22^,^23^. We suggest that this unique feature of near-surface MeHg maxima likely enhances MeHg exposure to the base of the Arctic marine food web and may explain the high MeHg levels of Arctic biota. Higher trophic level biota feeding in the marginal sea ice zone, including fish and marine mammals^2^, then bioaccumulate enhanced planktonic MeHg.

From our few profiles we posit that a combination of physical, biological and biogeochemical factors drives the shallow production of toxic bioaccumulating MeHg in the Arctic Ocean. Exactly how these factors, such as halocline stability, phytoplankton ecology, and nutrient biogeochemistry evolve with Arctic warming may determine future MeHg exposure to biota. Recent surface ocean and sea ice trends indicate stronger stratification, increased nitrogen limitation, and a subsequent reduction in phytoplankton size^34^. Small-sized phytoplankton is known to play a key role in marine MeHg dynamics^17^, because it sinks slower and boosts remineralization and MeHg production in the pycnocline waters. In parallel, small-sized plankton blooms occur deeper in the photic zone, closer to the MeHg maximum, which may further enhance biological uptake of MeHg. MeHg bioavailability to the base of the marine food web depends on a delicate balance between MeHg production and loss mechanisms, which are also affected by Arctic warming^12^. From our limited observations we therefore speculate that Arctic warming will likely lead to increased MeHg production and exposure and, in concert with the extension of the marginal sea ice zone, an extension of the MeHg production zone.

We hope that our findings will be guiding future Arctic Hg research, notably the international Arctic GEOTRACES multi-ship survey planned for summer 2015. More Hg speciation data is crucially needed along the open water - sea ice covered water gradient, with a particular focus on the marginal sea ice zone.

## Methods

The four profiles between the Siberian shelf/Laptev Sea and the North Pole (79–90°N) were sampled during the TransArc ARK XXVI/3 cruise^19^,^20^ in summer 2011 on the Research Vessel Polarstern. The 81 unfiltered samples were collected into pre-cleaned 250 mL PFA Teflon bottles (Savillex Purillex™) and acidified to 0.4 % (v:v) with double distilled HCl. Acidification rapidly converts dimethylmercury (DMHg) into monomethylmercury (MMHg)^35^, and we thus measured methylmercury (MeHg) as the sum of MMHg and DMHg. MeHg analysis in sea water is challenging due to the sub-picomolar levels, and the absence of certified reference materials or inter-comparison exercises^36^. For this study, we applied one of the best known reference methods, isotope dilution (ID), to a high sensitivity coupled gas chromatography – sector field ICP-MS (GC-SF-ICP-MS) method at the GET laboratory. MeHg and inorganic Hg species were extracted after derivatization, following previously published protocols^37^, that we further improved for ultra-trace levels. Briefly, enriched spikes of ^199^iHg and ^201^MeHg (ISC Science, Spain) were added to a 115 mL aliquot of the sea water samples, targeting optimal ratios of 8.46 for ^199^iHg_spike_/^202^iHg_sample_ and 4.25 for ^201^MeHg_spike_/^202^MeHg_sample_. The optimum spike to natural Hg isotope ratios was determined using the uncertainty magnification factor formula^38^. After 24h of equilibration, pH was adjusted to 3.9 with NH_3_ (ULTREX® II Ultrapure Reagent, J.T. Baker, USA) and a buffer solution made up with acetic acid (glacial, ULTREX® II Ultrapure Reagent, J.T. Baker, USA)/sodium acetate (J.T. Baker, USA). A solution of 1% (v:v) sodium tetra propyl borate (Merseburger Spezialchemikalien, Germany) was made up freshly, under cold conditions and avoiding contact with atmospheric oxygen. 1 mL of this solution was then added together with 200 μL hexane (Sigma Aldrich, USA). The glass bottles were hermetically sealed with Teflon-lined caps and vigorously shaken for 15 minutes. The organic phase was recovered and injected in the GC (Thermo Trace Ultra). The coupling to the high resolution ICP-MS (Thermo Element XR) and application of ultra-trace clean techniques allowed reaching detection limits as low as 0.001 pM. We then inter-compared for MeHg the ID-GC-SF-ICP-MS method to the established hydride generation - cryogenic trapping - cold vapor atomic fluorescence spectrometry (HG-CT-CV-AFS, AFS model: Tekran Model 2500, Canada) method at the IFREMER laboratory that produced two of the recent open ocean MeHg datasets for Southern Ocean^16^ and the Mediterranean Sea^17^. We measured the full depth profile of station 85°N and both methods gave similar results (r^2^ = 0.90; Fig. S3). tHg was measured independently as the given detection limit is given in a moles per volume unit. (pM) on a 35 mL aliquot following the USEPA 1631 method^39^ at the GET laboratory. Potassium bromide (Sigma Aldrich, USA) and Potassium Bromate (Sigma Aldrich, USA) were heated for 4 h at 250 °C to remove Hg traces before making up BrCl solution with freshly double-distilled HCl. We used a custom made semi-automatic single gold trap setup coupled to an cold vapor atomic fluorescence spectrometry (Brooks Rand Model III, USA), modified with mirrored quartz cuvette (Hellma Optics, Germany) to achieve a detection limit of 0.025 pM.

## Author Contributions

LEH, JES, DC, DP, CL and LL designed the study and developed the method to analyze ultra-low level MeHg concentrations in the sea water samples. BG, MN, BR and MRvdL participated on the cruise and provided samples. All authors contributed to manuscript preparation.

## Additional Information

**How to cite this article**: Heimbürger, L.E. *et al.* Shallow methylmercury production in the marginal sea ice zone of the central Arctic Ocean. *Sci. Rep.* **5**, 10318; doi: 10.1038/srep10318 (2015).

## Supplementary Material

Supplementary Information

## Figures and Tables

**Figure 1:**
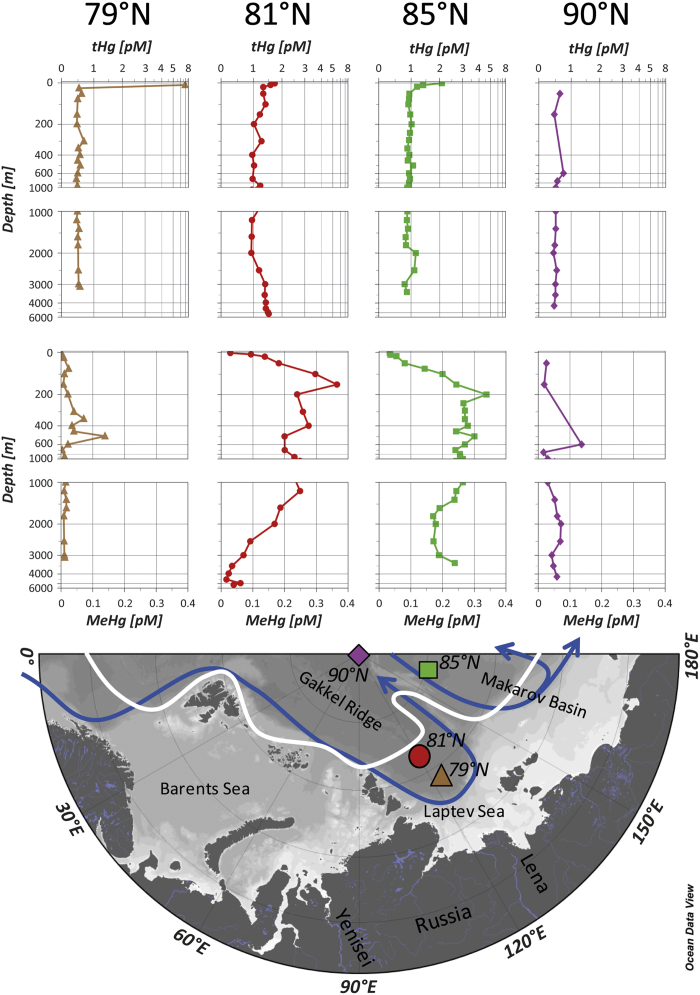
Total mercury (tHg) and methylmercury (MeHg) profiles in picomoles per litre (pM) at the coastal influenced open water Laptev Sea station (PS78/280:79°N; brown triangles), the open water Amundsen Basin station at the sea ice edge (PS78/273:81°N; red dots), the >75% sea ice covered Makarov Basin station (PS78/245:85°N; green squares), and the permanently sea ice-covered North Pole station (PS78/218:90°N, purple diamonds). The white line indicates the sea ice extent during the time of sampling. The blue line shows the general oceanic circulation of intermediate and Atlantic waters after Rudels, 2012 (Reference 27 in the manuscript). Map and plots were generated with Ocean Data View 4.0.
