# Supplementary material for: Shallow methylmercury production in the marginal sea ice zone of the central Arctic Ocean
Source: Sci Rep. 2015 May 20;5:10318. doi: 10.1038/srep10318 (PMC4438723; doi:10.1038/srep10318)
Supplement: Supplementary Information [file srep10318-s1.pdf]

Supplementary figures for:

Shallow methylmercury production in the marginal sea ice zone of the central Arctic Ocean

Lars-Eric Heimbürger[1*], Jeroen E. Sonke[1], Daniel Cossa[2], David Point[1], Christelle Lagane[1], Laure Laffont[1], Benjamin T. Galfond[3], Marcel Nicolaus[4], Benjamin Rabe[4] and Michiel Rutgers van der Loeff[4]

[1]Geosciences Environment Toulouse (GET), Observatoire Midi-Pyrénées (OMP), UMR CNRS 5563, UMR IRD 154, 14 avenue Edouard Belin, Université Paul Sabatier, 31400 Toulouse, France

[2]ISTerre, UJF, BP 53, 38041 Grenoble, France

[3]University Miami, Rosenstiel School Marine & Atmospheric Science, Miami, Florida 33149 USA

[4]Alfred-Wegener Institute for Polar and Marine Research, 27570 Bremerhaven, Germany

* *email: lars-eric@uni-bremen.de*

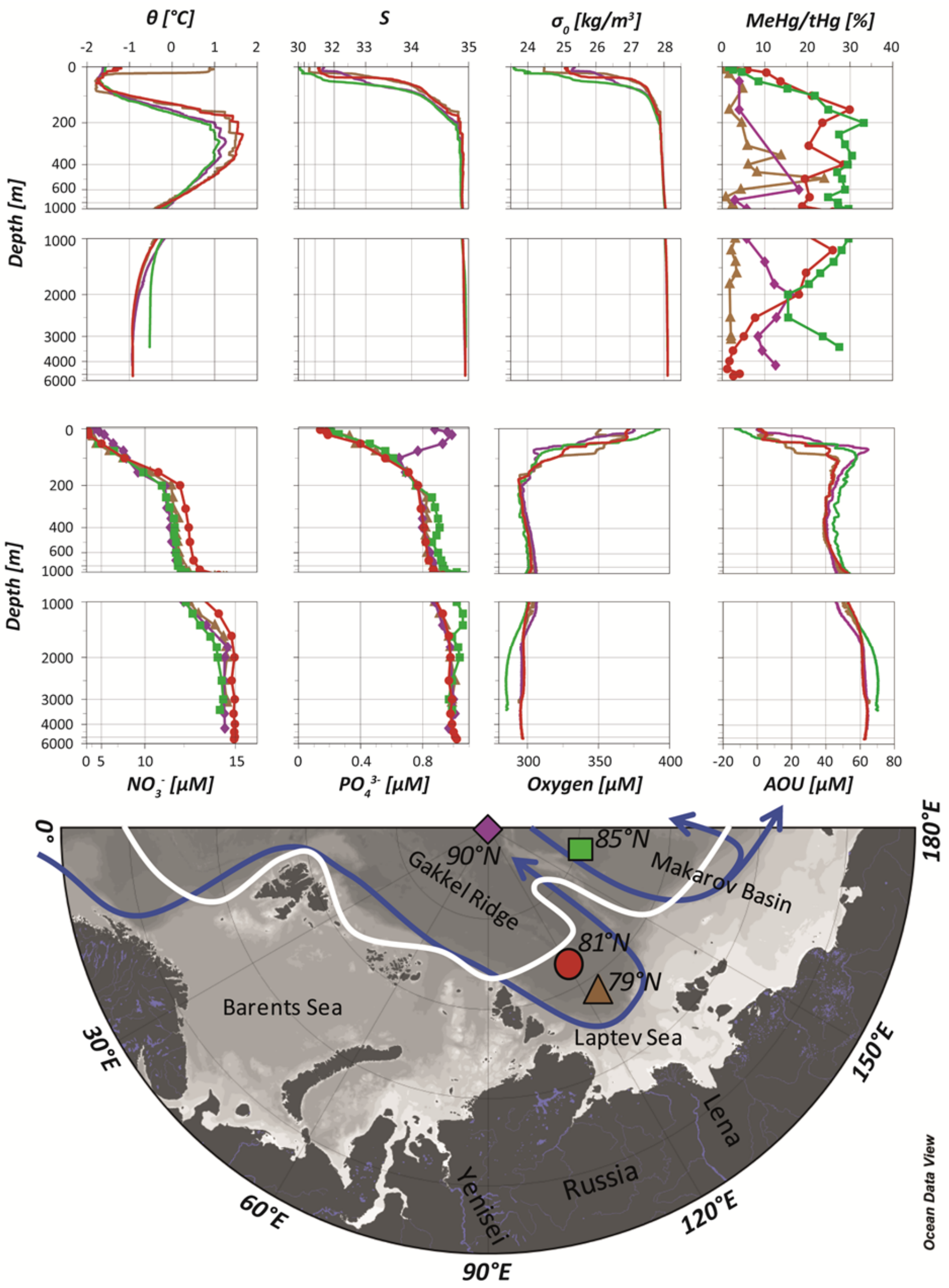

Fig. S1 : Potential temperature (Θ), salinity (S), density (σ_0), MeHg/tHg, nitrate (NO_3^-), phosphate (PO_4^{3-}), oxygen and apparent oxygen utilization (AOU) profiles at the coastal influenced open water Laptev Sea station (PS78/280:79°N ; brown triangles), the open water Amundsen Basin station at the sea ice edge (PS78/273:81°N; red dots), the > 75 % sea ice covered Makarov Basin station (PS78/245:85°N; green squares), and the permanently sea ice-covered North Pole station (PS78/218:90°N, purple diamonds). The red line indicates the sea ice extent during the time of sampling. The blue line shows the general oceanic circulation of intermediate and Atlantic waters (after Rudels, 2012; reference 27 in the manuscript). Original data for physical parameter and nutrients are available elsewhere: Schauer, U., Rabe, B. & Wisotzki, A. Physical oceanography during POLARSTERN cruise ARK-XXVI/3. Alfred Wegener Institute, Helmholtz Center for Polar and Marine Research, Bremerhaven: doi:10.1594/PANGAEA.774181 (2012) (Reference 19 in the manuscript). The nutrient data is available from Kattner, G. & Ludwichowski, K.-U. Inorganic nutrients measured on water bottle samples during POLARSTERN cruise ARK-XXVI/3 (TransArc). Alfred Wegener Institute, Helmholtz Center for Polar and Marine Research, Bremerhaven: doi:10.1594/PANGAEA.832164 (2014) (Reference 20 in the manuscript). Map and plots were generated with Ocean Data View 4.0.

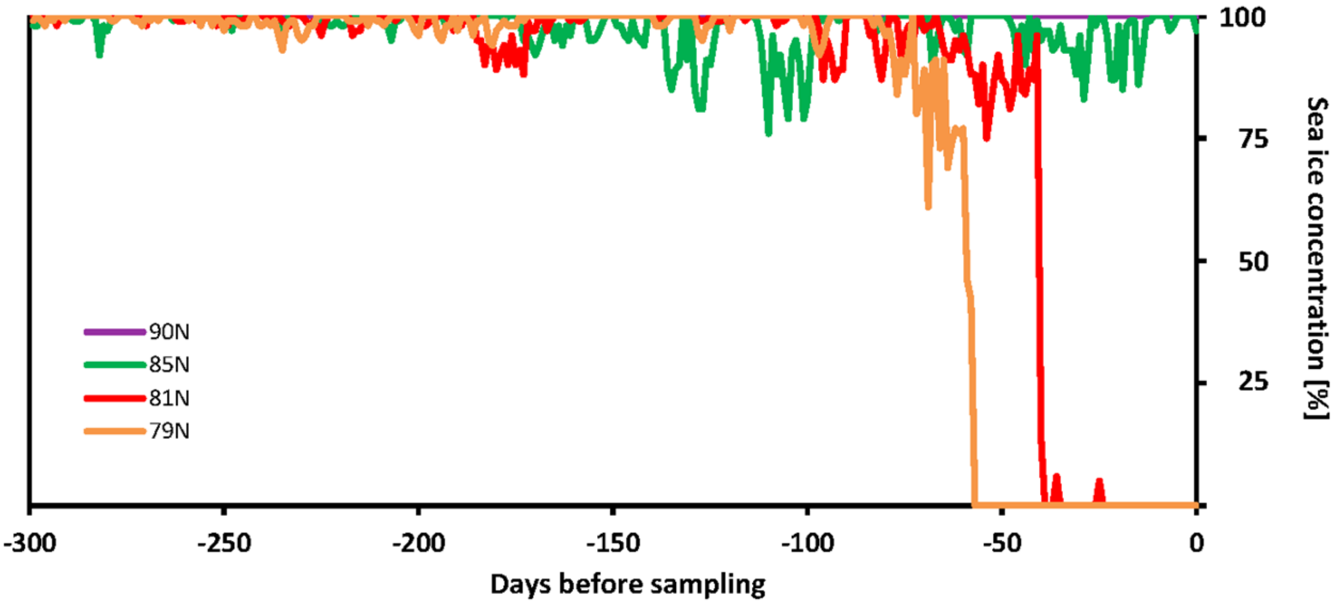

Fig. S2 : Sea ice concentration at the different measurement sites, as retrieved from the AMSR-E satellite, using the processor of University of Bremen on a 6.25 km grid. Original data is available at www.meereisportal.de . X-axis shows days before sampling. Station 79°N (brown) was ice free ~56 d before sampling, the station 81°N at the sea ice edge (red) was ice free 24 d before sampling, the Makarov Basin station 85°N (green) was always > 75 % sea ice covered. Satellite imagery is unable to provide observations at North Pole, but the ship board observations confirm the presence a full sea ice cover, dominated by multi-year ice at station 90°N (purple).

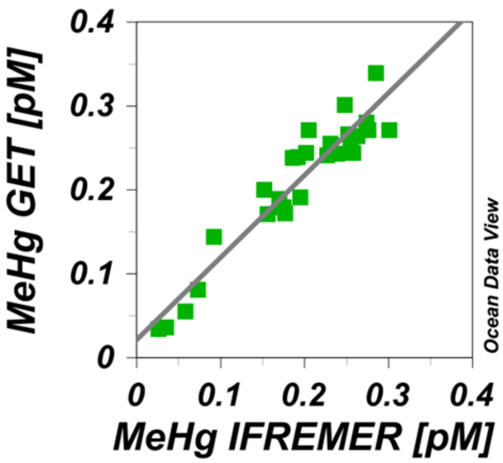

Fig. S3 : Comparison of MeHg determination *via* ID-GC-SF-ICP-MS (GET laboratory) and hydride generation - cryogenic trapping - cold vapor atomic fluorescence spectrometry HG-CT-CV-AFS (IFREMER laboratory) at station 85°N.

Least-squares line: MeHg GET = 0.98 MeHg IFREMER + 0.02, r^2= 0.90, n = 27. Plot was generated with Ocean Data View 4.0.
